# Intrahepatic Cholangiocarcinoma Staging and Classification Systems: An Updated Narrative Review

**DOI:** 10.1007/s12029-026-01594-9

**Published:** 2026-11-03

**Authors:** Andrea Marchese, Simone Conci, Laura Alaimo, Edoardo Poletto, Tommaso Campagnaro, Mario De Bellis, Andrea Ruzzenente

**Affiliations:** 1 Division of General and Hepatobiliary Surgery, Department of Surgical Sciences, Dentistry, Ginecology and Pediatrics, University of Verona, University Hospital G.B. Rossi, Verona, 37134 Italy; 2 Division of General and Hepatobiliary Surgery, Department of Surgical Sciences, Dentistry, Ginecology and Pediatrics, University of Verona Medical School, G.B. Rossi University Hospital, Piazzale L.A. Scuro, 10, Verona, 37134 Italy

**Keywords:** Intrahepatic cholangiocarcinoma, staging, classification, AJCC TNM, molecular profiling, precision oncology

## Abstract

**Background:**

Intrahepatic cholangiocarcinoma (ICC) is an aggressive primary liver malignancy characterized by increasing incdence, late diagnosis, and marked biological heterogeneity. This review summarizes ICC staging and classification systems, highlighting their clinical applications, strengths, limitations, and future development.

**Methods:**

Anatomical, histopathological, imaging-based, and molecular classifications were examined, with particular attention to prognostic discrimination, surgical planning, and treatment selection.

**Results:**

The AJCC eighth-edition TNM system remains the international standard and improves prognostic stratification through assessment of tumor size and number, vascular invasion, peritoneal perforation, extrahepatic extension, nodal involvement, and distant metastases. However, TNM staging does not fully capture biological heterogeneity. Macroscopic growth patterns and the World Health Organization distinction between large duct-type and small duct-type ICC provide complementary prognostic and surgical information. Institutional and morphology-based systems may refine postoperative risk assessment but have limited generalizability. Molecular profiling identifies clinically relevant subgroups characterized by FGFR2 fusions, IDH1/2 mutations, BAP1 and ARID1A alterations, or KRAS and TP53 mutations, with therapeutic implications.

**Conclusion:**

Contemporary ICC assessment should combine TNM stage with morphological, pathological, and molecular features. Future multidimensional frameworks incorporating tumor biology, treatment response, and circulating biomarkers may improve prognostication and personalized management, provided that international validation, accessibility, and clinical usability are maintained.

## Introduction

Intrahepatic cholangiocarcinoma (ICC) is a heterogeneous group of malignancies arising from the intrahepatic bile ducts, accounting for approximately 10–15% of all primary liver cancers, representing the second most common primary hepatic malignancy after hepatocellular carcinoma. [1] Over the past three decades, ICC incidence has increased worldwide, although reported trends vary geographically and are influenced by changes in disease classification [2, 3].

ICC is frequently diagnosed in advanced stages, with 60–70% of patients presenting unresectable disease [4, 5]. Its distinct clinical behavior and high biological heterogeneity need proper staging and classification systems to provide accurate prognostication and treatment selection criteria [6, 7].

Curative-intent liver resection remains the most effective therapeutic option achieving a five-year survival rate ranging from 20 to 40%. Prognosis is influenced by tumor characteristics (i.e. vascular invasion and multifocality), disease extent, and patient characteristics [6, 8]. Poor outcomes of ICC underscore the critical importance of accurate staging for prognostic counseling, therapeutic selection, and clinical trials design.

Staging systems are crucial in oncology, provide standardized tools for describing disease extent, facilitate communication within multidisciplinary teams, stratify patients for clinical trials and allow meaningful comparison of outcomes across institutions and time periods [4, 9]. The development of ICC specific staging systems has been challenged by disease’s low incidence, biological heterogeneity, evolving surgical approaches and limited understanding of molecular drivers [10, 11].

Early ICC staging systems were adapted from hepatocellular carcinoma or extrahepatic cholangiocarcinoma classifications, resulting in suboptimal prognostic discrimination. Recognition of ICC unique biological characteristics prompted development of ICC-specific staging systems. The American Joint Committee on Cancer (AJCC) introduced a dedicated ICC staging system in its 7th edition, followed by a substantial revision in the 8th edition based on large multinational validation studies [9, 10, 12]. Other institutional and international classification schemes have been proposed, emphasizing different prognostic variables. [11, 13–16].

Advances in understanding molecular characterization have had a disruptive impact on ICC management, recurrent genomic alterations including FGFR2 fusions, IDH1/2 mutations, BAP1 mutations, and alterations in chromatin remodeling genes have been identified [17–20]. These findings have enabled the development of targeted therapies demonstrating clinical efficacy in selected patients [21–24]. This therapeutic evolution may strengthen the rationale to integrate molecular features into staging and classification systems [25–28].

As treatment options expand with advances in surgical techniques, liver transplantation protocols, systemic therapies, and locoregional treatments, robust ICC staging systems is increasingly important for patient selection and assessment of treatment efficacy [29–36].

The aim of this review is to provide an updated overview of current ICC staging and classification systems, highlighting their strengths and limitations, discussing their potential clinical applications in guiding therapeutic decision-making. We analyze anatomical-pathological classifications based on growth patterns, TNM-based staging systems including the AJCC 8th edition, alternative prognostic models, and emerging molecular classifications [1, 9]. We discuss additional future perspectives for ICC staging, including integration of molecular biomarkers into staging systems.

## Histopathologic Classification

### Macroscopic Growth Patterns

The morphological heterogeneity of ICC has clinically relevant implications, as distinct macroscopic growth patterns are associated with different biological behaviors, treatment strategies, and outcomes [37–41]. The LiverCancer Study Group of Japan proposed a macroscopicclassificationof ICC into mass-forming (MF), periductal-infiltrating (PI), and intraductal growth (IG) types, with mixed patterns representing combinations of these forms [13]. Table 1.

**Table 1:** Morphological classification of intrahepatic cholangiocarcinoma

Growth type	Frequency (%)	Imaging characteristics	Histological features	5-y survival after resection (%)
Mass-forming	60–70	Well-defined mass, rim enhancement, delayed centripetal fill-in	Tubular/acinar architecture, desmoplastic stroma	30–40
Periductal-infiltrating	15–20	Bile duct wall thickening, ductal narrowing, upstream dilatation	Longitudinal spread along ducts, perineural invasion	10–20
Intraductal growth	5–10	Intraductal soft tissue/polypoid mass, ductal dilatation	Papillary/tubulopapillary growth, mucin production	50–65
Mixed types	10–15	Combined features	Mixed architectural patterns	20–35

The mass-forming type is the most prevalent subtype, accounting for approximately 60–70% of cases. It presents as discrete nodular masses within the hepatic parenchyma, typically demonstrating expansive growth with relatively well-defined margins on imaging and gross pathology [37, 38]. These tumors arise from smaller intrahepatic bile ducts and may be associated with background liver disease including cirrhosis, chronic hepatitis, or metabolic dysfunction. Histologically, MF ICCs usually exhibit tubular or acinar architecture with desmoplastic stroma and frequently demonstrate peripheral rim enhancement on arterial-phase imaging with progressive centripetal fill-in on delayed phases [42, 43].

The periductal-infiltrating type (PI), representing 15–20% of ICCs, is characterized by longitudinal spread pattern along bile ducts with ductal wall thickening and luminal narrowing without formation of a discrete mass [14, 38]. Diagnosis can be challenging, as imaging findings may be subtle and mimic benign biliary conditions. PI-ICC exhibits a strong propensity for extensive ductal spread and perineural invasion, leading to high rates of positive surgical margins despite extensive hepatectomy [44]. Prognosis is generally poor, with reported 5-year survival rates after resection frequently below 15–20% [13].

The intraductal growth type (IG), accounting for approximately 5–10% of ICCs, develops within bile duct lumens, often as intraductal papillary or tubulopapillary growth, sometimes with mucin production [37, 41]. These tumors may present with obstructive jaundice due to biliary obstruction despite limited tumor bulk. Many tumors historically classified as IG ICC fall within the spectrum of intraductal papillary neoplasm of the bile duct (IPNB) with or without associated invasive carcinoma. This should be considered when interpreting older outcome series [37, 41].

IG ICC often demonstrates more indolent biological behavior with lower rates of vascular invasion and lymph node metastasis [37]. When completely resected, long term outcomes are relatively favorable, with five-year survival rates exceeding 50–60%.

Mixed-type ICCs, accounting for 10–15% of cases, combines features of two or more growth patterns. Prognosis is generally intermediate or driven by the most aggressive component. [38] The most common mixed pattern combines mass-forming and periductal-infiltrating components.

Morphological classification has important implications for surgical planning and assessment of resectability [39]. Surgical resection with adequate margins offers favorable outcomes for MF tumors, whereas PI types frequenly require more extensive resection including bile duct resection and occasionally vascular reconstruction. Preoperative assessment of growth pattern can influence the surgical approach; meticulous assessment of bile duct margins and intraoperative frozen section analysis should be warranted for periductal-infiltrating tumors [42–44].

In recent studies morphological subtypes showed correlations with molecular alterations [38, 39]. PI ICC demonstrates higher frequencies of KRAS mutations and chromosomal instability, whereas MF types may show different mutational profiles. However, comprehensive genomic characterization across morphological subtypes remains incomplete, representing an important area for further investigation.The AJCC 8th edition does not include macroscopic growth pattern as an independent staging criterion. Periductal invasion, classified as T4 in the 7th edition, was removed as a separate category criterion [9]. However, integration of morphological classification within TNM staging can provide complementary prognostic information. Several studies demonstrate that growth pattern provides prognostic information independent of TNM stage [13]. Future staging systems may benefit from formal integration of growth pattern to enhance prognostic accuracy.

### Histological Subtypes

The 2019 World Health Organization (WHO) Classification of Tumors of the Digestive System introduced a clinically significant subdivision of ICC into two distinct subtypes based on presumed cell of origin and anatomical derivation: large duct-type ICC and small duct-type ICC [45]. The classification is based on fundamental differences in histogenesis, morphology, molecular profile, and clinical behavior between the two subtypes, which provides a framework that complements macroscopic growth pattern classifications [37, 39–41].

Large duct-type ICC is thought to arise from larger intrahepatic bile ducts [40, 45] Histologically, these tumors are characterized by mucin-producing columnar or cuboidal cells arranged in tubular or papillary patterns, frequently accompanied by abundant desmoplastic stroma [37, 39]. Large duct-type ICC commonly presents as a PI ICC or mixed-type ICC on macroscopic examination [41]. From a molecular standpoint, this subtype is associated with higher rates of KRAS mutations, TP53 alterations, and chromosomal instability, patterns reminiscent of pancreatobiliary adenocarcinomas [38, 39, 46]. Clinically, large duct-type ICC tends to behave more aggressively, with higher propensity for lymph node metastasis, perineural invasion, and positive surgical margins, contributing to worse overall prognosis [40].

Small duct-type ICC, conversely, is thought to originate from the peripheral intrahepatic ductules, canals of Hering, or hepatic progenitor cells [40, 45]. Histologically, these tumors typically exhibit non-mucin-producing cuboidal cells forming small anastomosing tubules or cords embedded in a fibrotic but less prominent stroma. [37, 39] Macroscopically, small duct-type ICC most commonly presents as a MF ICC [41]. The molecular landscape of small duct-type ICC is characterized by a higher prevalence of FGFR2 fusions or rearrangements, IDH1/2 mutations, BAP1 alterations, and ARID1A mutations [17–19, 38]. Among these alterations, FGFR2 fusions or rearrangements and IDH1 mutations have established therapeutic relevance in advanced disease [21, 22]. Small duct-type ICC generally exhibits a comparatively more indolent behavior, with lower rates of nodal metastasis and a somewhat better prognosis after curative resection [38, 40].

The clinical relevance of the WHO large duct versus small duct dichotomy extends beyond histological categorization. The two subtypes show distinct epidemiological associations: large duct-type ICC is more frequently linked to biliary tract pathology including primary sclerosing cholangitis, hepatolithiasis, and biliary cysts, whereas small duct-type ICC shows stronger associations with chronic hepatitis B and C virus infection, hepatic steatosis, and metabolic liver disease [38, 40, 46]. Furthermore, the differential molecular profiles of the two subtypes support the rationale for subtype-specific treatment strategies, particularly in the advanced disease setting where molecularly targeted therapies are increasingly employed [21, 22, 25]. Despite its conceptual and clinical value, routine implementation of the WHO large duct versus small duct classification in pathological practice remains variable, partly due to overlapping morphological features in a subset of cases and limited interobserver reproducibility in borderline tumors [39, 41, 45]. Nonetheless, the WHO classification represents an important step toward a more biologically integrated approach to ICC characterization, and its incorporation into future staging frameworks and clinical trial stratification is strongly advocated [25, 45, 47].

## Clinical and Macroscopic Staging

### AJCC TNM Staging Systems (7th and 8th Editions)

The American Joint Committee on Cancer (AJCC) TNM staging system represents the most widely adopted ICC classification, providing standardized criteria to describe the extent of disease. The development of ICC-specific AJCC staging has undergone significant evolution, reflecting accumulating clinical evidence.

Before 2010, ICC was staged using hepatocellular carcinoma or extrahepatic cholangiocarcinoma systems, which inadequately captured ICC-specific prognostic factors. The AJCC 7th edition introduced the first ICC-specific staging system, representing a major advance in standardizing ICC classification [10]. Thissystem was informed by Nathan et al.''s population-based analysis of 598 surgically treated patients in the SEER database, which highlighted tumor multiplicity and vascular invasion as prognostic factors [10, 12]. However, the 7th edition demonstrated suboptimal prognostic discrimination, particularly for early-stage disease, and uncertainty regarding biological significance of multiple tumors.

The AJCC 8th edition introduced substantial revisions [9]. The 8th edition T-stage definitions include solitary tumors without vascular invasion (T1), solitary tumors with vascular invasion or multiple tumors regardless of vascular invasion (T2), tumors perforating the visceral peritoneum (T3), and tumors with extrahepatic extension including direct invasion of adjacent organs (T4). Within T1 stage, subdivision into T1a (solitary tumor ≤ 5 cm without vascular invasion) and T1b (solitary tumor > 5 cm without vascular invasion) recognizes tumor size as prognostic factor [9].

T2 encompasses solitary tumors with vascular invasion and multifocal tumors with or without vascular invasion, reflecting consistent evidence linking both features to adverse outcomes [12, 48]. T3 includes tumors perforating the visceral peritoneum, associated with increased risk of peritoneal dissemination, while T4 includes tumors with direct invasion of adjacent structures, which are associated with particularly poor prognosis [9].

Regional lymph node staging is binary (N0 vs. N1), with any regional nodal metastasis conferring N1 status [9]. Although nodal burden and lymph node ratio may further stratify risk, the binary approach reflects practicality and the dominant adverse impact of nodal involvement [9, 49–51]. Detection of distant metastases generally preclude standard curative-intent surgery and changes the treatment approach toward systemic therapy and supportive care.

Stage grouping combines TNM components into stages I–IV. Validation studies consistently demonstrate improved prognostic discrimination for the AJCC 8th edition compared with the 7th edition, particularly in early-stage disease [8, 12, 15, 52]. Nonetheless, heterogeneity persists, particularly within stage II, highlighting the need for additional prognostic variables beyond anatomical extent [48].

Despite it is widely used, the AJCC 8th edition has recognized limitations. The system does not include morphological growth patterns, tumor grade, molecular features, or performance status, which could contribute prognostic information. The binary N-staging may oversimplify prognostic heterogeneity among node-positive patients. Future editions will likely consider integration of additional variables to enhance prognostic accuracy and clinical utility.

### Alternative and Modified Staging Systems

Nathan et al. developed an ICC-specific staging proposal using 598 surgically treated patients from the SEER database. The proposal incorporated tumor number, vascular invasion, nodal status, and distant metastasis, while omitting tumor size. This proposal contributed to the development of ICC-specific staging [10].

Okabayashi et al. proposed a staging systembased on 60 patients undergoing curative-intent resection for mass-forming ICC. Stage I comprised solitary tumors without vascular invasion; stage II, solitary tumors with vascular invasion; stage IIIA, multiple tumors with or without vascular invasion; stage IIIB, regional nodal metastases; and stage IV, distant metastases. The small derivation cohort and restriction to mass-forming limit generalizability [13].

The Liver Cancer Study Group of Japan developed an ICC-specific staging framework. Sakamoto et al. subsequently proposed a revised system using nationwide surgical data, incorporating tumor number, tumor size with a 2 cm cutoff, arterial invasion, and major biliary invasion. This proposal provides an alternative framework for prognostic stratification after resection [15, 16].

Several studies have compared the performances of different staging systems within the same patient cohorts, providing insights into relative strengths [6, 15, 52]. These comparisons employed concordance indices (C-index) or area under receiver operating characteristic curves (AUC) to quantify their discriminatory ability. Comparison of the AJCC 7th and 8th editions have shown cohort-dependent differences in prognostic stratification, without a consistent marked improvement in overall discrimination [9, 15]. Systems incorporating lymph node status generally demonstrate superior discrimination compared to those relying solely on T-stage factors [12, 50, 51].

Interestingly, some studies suggest that combined models incorporating elements from multiple systems may optimize prognostic accuracy [52, 53]. For instance, adding morphological growth pattern to AJCC stage or incorporating margin status to TNM classification may incrementally improve C-indices, representing meaningful enhancement in prognostic discrimination. These observations support development of multidimensional staging frameworks integrating anatomical, pathological, and potentially molecular variables. Table 2.

**Table 2:** Comparison of major ICC staging systems

Feature	AJCC 8th edition	AJCC 7th edition	Okabayashi system
Primary Focus	Anatomical extent	Anatomical extent	Morphology + extent
Tumor Number	T2 (multiple tumors)	T2b (multiple tumors)	Incorporated
Vascular Invasion	T2 (with VI)	T2a (with VI)	Stage II criterion
Peritoneal Perforation	T3	T3	Not specifically addressed
Lymph Node Status	Binary (N0/N1)	Binary (N0/N1)	Stage III if positive
Morphological Type	Not incorporated	PI classified as T4	Primary criterion
Resection Margin	Not incorporated	Not incorporated	Not specifically addressed
Validation	International	International	Japanese cohort (small)

## Molecular Cluster Classifications

The molecular era has revolutionized understanding of ICC biology, revealed remarkable genomic heterogeneity and identified therapeutic targets [17–20, 54–56]. Comprehensive genomic profiling studies have characterized the mutations of ICC, enabling development of molecular classification schemes that complement anatomical staging systems.

### Most Frequent Genomic Mutations and Alterations

Intrahepatic cholangiocarcinoma is characterized by marked genomic heterogeneity with a distinct mutational landscape that differs substantially from other gastrointestinal malignancies [17, 18, 54–56]. Comprehensive next-generation sequencing studies have identified a set of recurrently mutated genes that define biologically and clinically relevant subgroups. Among the most clinically actionable alterations, FGFR2 fusions or rearrangements are detected in approximately 10–17% of ICC cases, occurring predominantly in small duct-type tumors and in Western populations [46, 57]. These alterations are now targeted by approved agents including pemigatinib and futibatinib, that have demonstrated antitumorali activity in previously treated FGFR2-fusion or rearrangement-positive cholangiocarcinoma [21]. IDH1 and IDH2 mutations occur in a subset of ICCs. The established therapeutic evidence for ivosidenib applies specifically to IDH1-mutant cholangiocarcinoma [22, 58]. BAP1 mutations and ARID1A alterations are found in approximately 15–25% and 10–20% of cases respectively, and are enriched in the small duct subtype, contributing to epigenetic dysregulation and chromatin remodeling defects without yet having a directly matched targeted therapy [19, 74]. In contrast, KRAS mutations, occurring in 8–25% of ICC cases, and TP53 alterations are more prevalent in large duct-type tumors and are consistently associated with aggressive tumor behavior and worse overall survival across molecular classification studies [17, 59, 74]. Other less frequent but potentially actionable alterations include BRAF V600E mutations (approximately 5%), HER2 amplifications (approximately 4%), NTRK fusions, and high microsatellite instability or mismatch repair deficiency, the latter being responsive to immune checkpoint inhibitors [18, 47, 55]. This diverse mutational landscape underscores the need for comprehensive molecular profiling as a standard component of ICC workup, particularly in patients with unresectable or metastatic disease, to guide selection of targeted and immunotherapeutic strategies.

### Molecular Cluster Classification Proposal

Beyond individual mutational alterations, multi-omics classification studies have applied unsupervised clustering approaches integrating transcriptomic, proteomic, epigenomic, and copy number data to define biologically coherent ICC subtypes with prognostic significance [60, 74]. A recent systematic review and meta-analysis encompassing nine multi-omics studies and 910 ICC patients demonstrated that molecular cluster classification consistently stratifies patients into at least two prognostically distinct groups across different platforms and cohorts [61]. Patients in the good-prognosis molecular cluster showed a mean 5-year overall survival of 63.3%, compared with 19.6% in the poor-prognosis cluster, with a pooled hazard ratio of 2.06 (95%CI 1.67–2.53) for the bad-prognosis subtype [61]. Molecular classifications identify prognostically distinct but partly overlapping subgroups. The meaning of inflammatory or immune-related signatures varies across classification schemas. In Sia et al., the proliferation class had worse outcomes than the inflammation class [60]. The tumor microenvironment (TME) plays a central role in determining ICC molecular subtype and prognosis [62, 63]. Cancer-associated fibroblasts (CAFs) and tumor-associated macrophages produce paracrine and vascular factors that promote desmoplastic stroma formation, pro-tumorigenic signaling, and immune evasion, all features enriched in the mesenchymal ICC subtype associated with poor outcomes [63, 64]. Conversely, immune-enriched subtypes characterized by active antitumor immune infiltration, including T cells, B cells, and immune checkpoint expression, have been linked to comparatively better survival and potential responsiveness to immune checkpoint inhibitors [61, 63]. An ambivalent immune profile, combining both immune-stimulating and immune-suppressive signals, has been described in subtypes with intermediate prognosis [61]. The integration of clinical, pathological, and molecular data across these classification systems highlights that KRAS mutation, large duct histological type, inflammatory and mesenchymal gene expression enrichment, and advanced TNM stage are consistently associated with a poor prognosis, whereas FGFR2 and IDH1/2 alterations characterize biologically distinct subsets, but their prognostic implications should not be equated with their therapeutic actionability [47, 60, 61, 74]. Table 3.

**Table 3:** Cluster Classification of ICC on Good-Prognosis Subtype and Poor-Prognosis Subtype

Feature	Good-Prognosis subtype	Poor-Prognosis subtype
Key mutations	FGFR2 fusions/rearrangements, IDH1/2 mutations	KRAS mutations, TP53 alterations, SMAD4, BRAF V600E
Gene expression signature	Immune-enriched, metabolic pathways	Inflammatory (classification-dependent definition) mesenchymal, proliferative pathways
Histological subtype	Small duct-type (predominant)	Large duct-type (predominant)
Macroscopic growth pattern	Mass-forming (predominant)	Periductal-infiltrating or mixed
Tumor microenvironment	Immune-inflamed; active T-cell/B-cell infiltration; immune checkpoint expression	Desmoplastic stroma; CAF- and TAM-enriched; immune-excluded or immune-desert
Pathological features	Lower rates of vascular and perineural invasion; lower nodal metastasis rate	Higher rates of perineural invasion, vascular invasion, and nodal metastasis
Clinical/lab markers	Normal/low CEA; lower CA19-9; early TNM stage (I–II)	Elevated CEA (> 5 ng/mL) and CA19-9; advanced TNM stage (III–IV)
Survival outcomes	Mean 3-yr OS 70.2%; mean 5-yr OS 63.3%	Mean 3-yr OS 25.7%; mean 5-yr OS 19.6%
Available targeted therapies	FGFR inhibitors (pemigatinib, futibatinib); IDH1 inhibitor (ivosidenib); immune checkpoint inhibitors (MSI-H/dMMR)	Gemcitabine-based chemotherapy; clinical trials (KRAS/BRAF inhibitors under investigation)
Aetiology associations	HBV/HCV infection; metabolic liver disease; hepatic steatosis	PSC; hepatolithiasis; biliary cysts; HBV (in some cohorts)

*CAF* cancer-associated fibroblast, *TAM* tumor-associated macrophage, *CEA* carcinoembryonic antigen, *CA19-9* carbohydrate antigen 19 − 9, *OS* overall survival, *PSC* primary sclerosing cholangitis, *HBV* hepatitis B virus, *HCV* hepatitis C virus, *MSI-H* microsatellite instability-high, *dMMR *deficient mismatch repair

### Integration of Molecular data into Clinical Staging

While molecular features increasingly inform treatment decisions through precision medicine approaches, formal integration into anatomical staging systems remains limited [9, 25, 47]. The AJCC 8th edition staging manual acknowledges the importance of molecular markers but does not incorporate them into stage groupings, citing need for further validation, standardization of testing platforms, and evidence demonstrating that molecular features add independent prognostic information beyond anatomical stage [9].

Future iterations of staging systems may incorporate molecular markers, potentially creating integrated prognostic algorithms combining anatomical extent, molecular subtype, and clinical factors [25, 47]. Such multidimensional staging would enable more accurate prognostication and inform personalized treatment strategies. However, challenges include ensuring broad access to molecular testing, establishing which molecular features merit inclusion, and maintaining system simplicity for practical implementation [47, 56].

### Strengths and Limitations of Different Systems

The AJCC TNM staging system offers several advantages including international standardization, widespread adoption facilitating outcome comparisons across institutions and clinical trials, relative simplicity with clearly defined categories, and extensive validation in diverse patient populations [8, 9, 12, 52]. The 8th edition improvements in prognostic discrimination represent meaningful advances. However, AJCC staging does not incorporate morphological growth patterns, tumor grade, molecular features, or patient performance status, which influence outcomes [9, 37–39]. Additionally, the binary N-staging may oversimplify prognostic heterogeneity among node-positive patients [49–53, 65, 66]. Alternative staging proposals, including the Okabayashi and LCSGJ systems, were developed in specific surgical populations and require consideration of their derivation cohorts and external validity [13, 15].

Molecular classification schemes reflect cutting-edge understanding of ICC biology and enable precision medicine approaches but face barriers to widespread implementation including cost, technical requirements, lack of standardization, and insufficient outcome data in large cohorts [17, 18, 25, 47, 56]. As molecular testing becomes routine and targeted therapies proliferate, molecular features will increasingly complement anatomical staging [21, 22, 26, 67].

### Recommendations for Clinical Practice

For clinical practice, a multifaceted approach incorporating elements from multiple classification systems optimizes patient assessment [1, 4, 68]. Initial diagnostic workup should establish anatomical extent using AJCC TNM staging as the foundation for communication and treatment planning[9]. Preoperative imaging should characterize morphological growth pattern, as this influences surgical approach and provides complementary prognostic information [9, 37, 42–44]. These pathological findings support AJCC staging and may contribute to post-resection prognostic models [9, 52, 65, 66]. Post-resection prognostication benefits from multivariable nomograms incorporating pathological findings to provide individualized survival estimates and guide adjuvant therapy discussions [52, 65, 66, 69, 71].

For advanced disease, molecular testing should be pursued to identify targetable alterations including FGFR2 fusions, IDH1/2 mutations, and other actionable genomic alterations. [21, 22, 25, 47, 67, 68] Molecular profiling should be performed before or during first-line systemic therapy in advanced ICC to inform subsequent treatment selection. 

### Treatment Planning Implications

Treatment planning should integrate TNM stage with technical resectability, liver reserve, performance status, and multidisciplinary assessment [1, 31, 65, 66, 68, 70, 71]. AJCC Stage I-II disease without adverse features (periductal infiltration, poor differentiation, elevated tumor markers) represents optimal candidates for upfront resection [8, 9]. Stage II disease with high-risk features or Stage IIIA disease might benefit from neoadjuvant therapy, though evidence remains limited and institutional practices vary. Stage IIIB disease includes T4N0M0 and any T N1M0 disease [9]. Neoadjuvant therapy may be considered in selected high-risk or borderline-resectable cases, but is not dictated by stage alone. Adjuvant capecitabine should be offered to appropriate patients after resection, rather than be restricted to node-positive disease [29, 71, 72]. Stage IV disease is generally managed with systemic therapy, while surgery after response is exceptional and requires individualized multidisciplinary consideration [30–33, 67, 68, 73].

Beyond stage, morphological growth pattern influences surgical strategy [13, 37]. For mass-forming ICC, the surgical objective is an R0 resection with an adequate future liver remnant. The extent and anatomical nature of resection should be individualized. Periductal-infiltrating tumors may require bile duct resection with frozen section margin assessment and regional lymph node assessment [12, 44]. The presence of major vascular invasion or large tumor size might prompt discussion of neoadjuvant therapy or vascular reconstruction [10, 12, 48].

Molecular features increasingly guide systemic therapy selection [21, 22, 25, 26, 57, 74, 75]. Patients with previously treated advanced ICC harboring FGFR2 fusions or rearrangements or IDH1 mutations may be candidates for matched therapy, according to the specific alteration and applicable treatment recommendations [21–24, 58]. Patients with mismatch repair deficiency or high tumor mutational burden may benefit from immune checkpoint inhibitors [33, 63]. As additional targeted therapies emerge, molecular profiling will become standard for advanced ICC [25, 26, 47].

## Future Perspectives

The field of ICC staging and classification continues to evolve rapidly, driven by expanding therapeutic options, improving molecular understanding, and accumulating outcome data from large patient cohorts [1, 17, 18, 28, 56]. Several important trends and developments will shape future classification systems.

Future staging systems will likely integrate molecular markers with anatomical extent to create multidimensional prognostic frameworks [25, 47, 56]. Hybrid systems combining TNM stage, morphological growth pattern, key molecular alterations (FGFR2, IDH1/2, TP53, KRAS), and clinical factors (performance status, liver function) may optimize prognostic accuracy [17, 53, 54, 56, 64]. The challenge lies in determining which molecular features add sufficient independent prognostic value to merit inclusion while maintaining system usability. Machine learning approaches analyzing large datasets may identify optimal variable combinations for prognostic algorithms.

Current staging systems assess disease at single timepoints, yet tumor biology is dynamic and treatment response provides crucial prognostic information [29–33]. Future frameworks may incorporate baseline staging plus response to initial therapy (such as neoadjuvant treatment response grade or radiological response assessment) to create dynamic risk stratification. Patients with significant response to therapy may have prognoses more favorable than baseline stage alone would suggest. Integration of treatment response into prognostic models represents conceptual shift toward adaptive staging paradigms.

The tumor microenvironment, particularly immune cell infiltration patterns and checkpoint molecule expression, influences ICC biology and treatment response [62, 64].

 Immune-related subtypes have shown prognostic associations, but their role as predictive biomarkers for immunotherapy requires prospective clinical validation [33, 63]. Future classification systems may incorporate immunological features, particularly as immune checkpoint inhibitors and combination immunotherapy approaches demonstrate efficacy in selected ICC populations [33, 63, 73].

As precision medicine advances, staging systems may become increasingly personalized, incorporating individual patient genomic features, circulating tumor DNA dynamics, metabolic profiles, and other biomarke [25, 26, 47]. Liquid biopsy approaches detecting circulating tumor DNA may enable non-invasive disease monitoring and early recurrence detection, informing adaptive treatment strategies. Integration of dynamic biomarker monitoring into clinical algorithms represents frontier of personalized oncology.

The proliferation of staging systems, while reflecting scientific progress, creates challenges for outcome comparison and clinical trial design [1, 4, 9, 68]. International consensus efforts bringing together major hepatobiliary societies to develop unified staging frameworks would benefit the field. Such efforts should balance comprehensive disease characterization with practical simplicity, ensure global applicability across healthcare settings, and incorporate flexible frameworks accommodating emerging biomarkers.

## Conclusions

Intrahepatic cholangiocarcinoma staging and classification underwent substantial evolution over recent decades, progressing from borrowed systems to ICC-specific frameworks incorporating growing understanding of disease biology. The AJCC 8th edition TNM system represents the current international standard, demonstrating improved prognostic discrimination compared to earlier iterations. However, no single system perfectly captures ICC’s biological heterogeneity, and contemporary practice benefits from multifaceted assessment incorporating anatomical staging, morphological classification, pathological features, and increasingly, molecular characteristics. 

Alternative systems including institutional models and morphology-based classifications provide complementary prognostic information valuable in specific clinical contexts. The molecular era has revealed genomic heterogeneity with therapeutic implications, prompting efforts to incorporate molecular features into staging paradigms. 

As treatment options expand, future staging systems will likely evolve toward multidimensional systems integrating anatomical, molecular, and clinical features through sophisticated prognostic algorithms. The challenge lies in balancing comprehensiveness with usability, ensuring broad applicability while accommodating emerging biomarkers, and maintaining international standardization while allowing regional customization.

Ultimately, staging systems serve as tools to guide clinical decision-making, facilitate communication, and enable meaningful outcome comparisons. The optimal system depends on clinical context: the AJCC TNM system for standardized international communication and clinical trial design, morphology-based systems for surgical planning, validated post-resection prognostic models for post-resection prognostication, and molecular classification for precision therapy selection. 

Further investigations through multi-institutional collaborations, prospective registries, and genomic profiling initiatives will further refine ICC classification. The goal remains improving outcomes through accurate prognostication, personalized treatment selection, and development of more effective therapeutic strategies for this aggressive disease.

## Data Availability

No datasets were generated or analysed during the current study.
